# Cadiology intensive care in patients with out-of-hospital cardiac arrest or cardiogenic shock

**DOI:** 10.1016/j.resplu.2025.101116

**Published:** 2025-09-30

**Authors:** Vera Garcheva, Tobias J. Pfeffer, Johann Bauersachs, Andreas Schäfer

**Affiliations:** Cardiac Arrest Centre, Department of Cardiology and Angiology, Hannover Medical School, Hannover, Germany

**Keywords:** Cardiac arrest, Cardiopulmonary resuscitation, Therapeutic hypothermia, Cardiogenic shock, Intensive care

## Abstract

**Background:**

Despite advances in therapy, mortality remains high after out-of-hospital cardiac arrest (OHCA) and cardiogenic shock (CS). While recent trials have improved CS care, OHCA management appears to have stagnated following neutral or negative results.

**Objectives:**

To evaluate the Hannover Cardiac Resuscitation Algorithm (HaCRA) for standardized early diagnostic and therapeutic management of OHCA and CS patients prior to intensive care admission.

**Methods:**

All OHCA and CS patients admitted under HaCRA underwent structured evaluation for ventilatory and circulatory support, including non-invasive imaging, cardiac catheterization with revascularization, mechanical circulatory support, therapeutic hypothermia, and invasive haemodynamic monitoring. A cardiology intensive care team supervised care from admission to intensive care.

**Results:**

A total of 946 OHCA and 506 CS patients were treated. Mechanical circulatory support was required in 21 % of OHCA patients. Among CS patients receiving a micro-axial flow pump, 49 % had been resuscitated beforehand. OHCA mortality was 44 % overall, 33 % in shockable rhythms, and 61 % in non-shockable rhythms. Patients meeting inclusion criteria of the *targeted temperature management (TTM)*-trial had a mortality rate of 23 % with predominantly good neurological outcomes. CS patients requiring circulatory support had 52 % mortality, ranging from 35 % with micro-axial flow pump support to 59 % with biventricular support.

**Conclusions:**

Implementation of HaCRA, coordinated by cardiology consultants trained in both interventional cardiology and intensive care, standardizes the management of OHCA and CS and may improve outcomes in these critically ill populations.

## Graphical abstract

**Figure:**
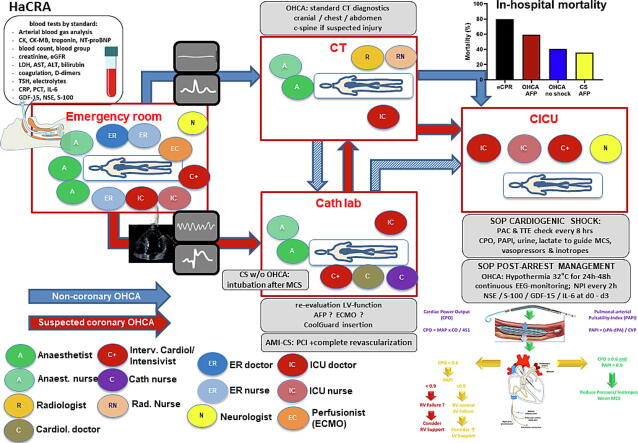

## Introduction

### Background

Implementation of HaCRA, coordinated by cardiology consultants trained in both interventional cardiology and intensive care, standardizes the management of OHCA and CS and may improve outcomes in these critically ill populations.^1^, ^2^ OHCA and CS frequently overlap, as acute myocardial infarction (AMI) is a common trigger for both. Despite advances in therapy, mortality remains ≥ 50 % in both entities across clinical trials^3^, ^4^, ^5^, ^6^ as well as registries.^7^, ^8^, ^9^, ^10^, ^11^ Standardized protocols may accelerate diagnosis, streamline treatment, and improve outcomes.

To address this need, we developed the Hannover Cardiac Resuscitation Algorithm (HaCRA), an interdisciplinary pathway for OHCA and CS management.^12^ HaCRA integrates recommendations from the German Resuscitation Council^13^ and international guidelines,^14^, ^15^, ^16^ emphasizing rapid identification of coronary stenosis and ischemia, impaired end-organ perfusion, trauma, and resuscitation-related injuries. Patients without return of spontaneous circulation (ROSC) are considered for extracorporeal cardiopulmonary resuscitation (eCPR),^17^ while those with profound CS undergo stabilization with a micro-axial flow pump (AFP),^5^, ^18^ supplemented by veno-arterial extracorporeal membrane oxygenation (vaECMO) in biventricular failure.^19^, ^20^ Definitive coronary angiography and complete revascularisation immediately follow haemodynamically stabilization.^21^ The initial diagnostic approach to the patient including early assessment and treatment is shown in the Central Illustration.

**Central Illustration:**
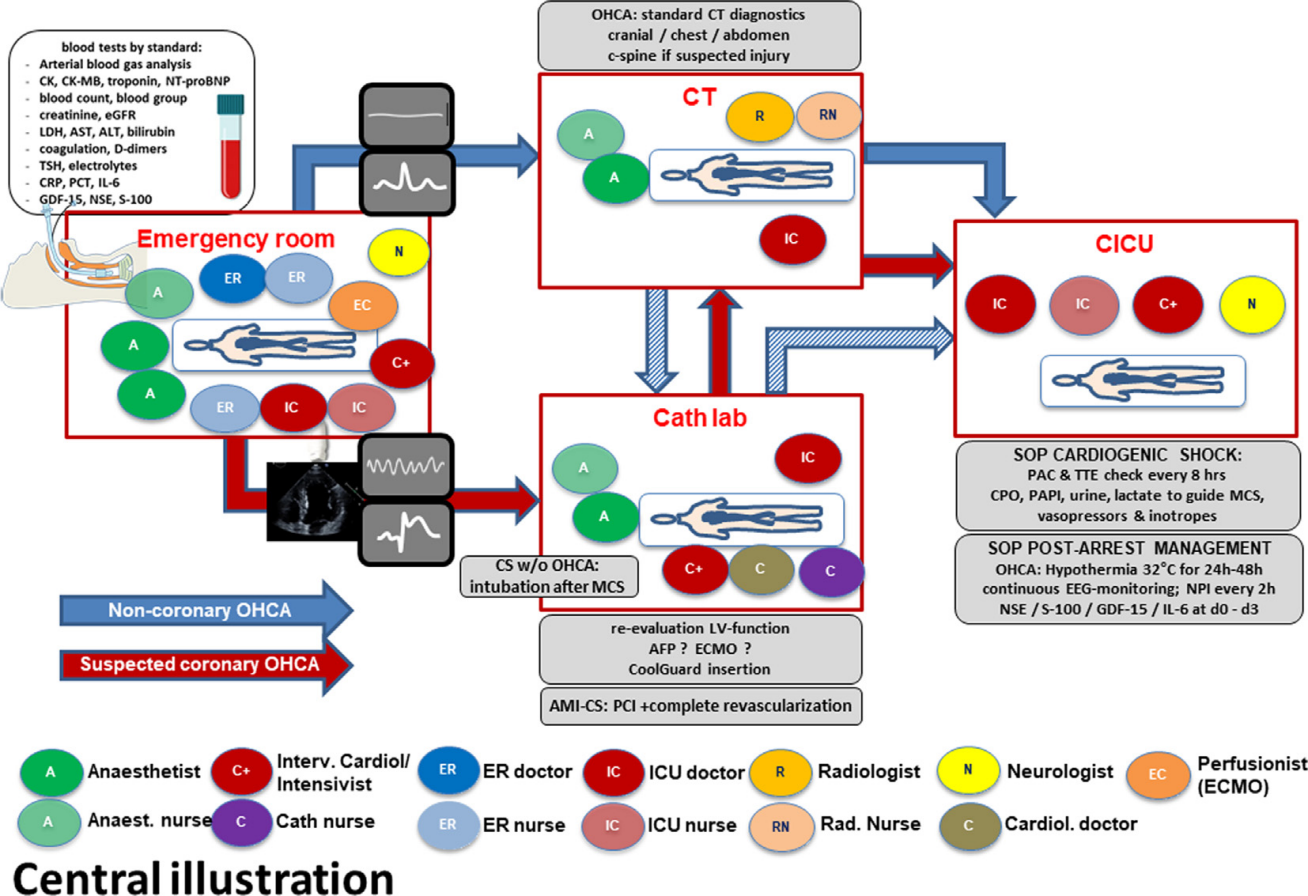
The Interdisciplinary algorithm – the Hannover Cardiac Resuscitation Algorithm (HaCRA) – is a treatment algorithm delineating diagnostic and interventional responsibilities of all participating professions (ER nurses, cardiology, anaesthesiology, radiology, neurology, intensive care) in the work-up of any out-of-hospital cardiac arrest or cardiogenic shock patient admitted at Hannover Medical School. AFP – axial flow-pump, AMI-CS – acute myocardial infarction-related cardiogenic shock, CICU – Cardiology intensive care unit, CPO – cardiac power output, CT – computed tomography, ECG – electro-cardiogram, EEG – electro-encephalogram, ECMO – extra-corporal membrane oxygenation, ER – emergency room, GDF-15 – Growth-derived factor 15, ICB – intracranial bleeding, IL-6 – Interleukin 6, LV – left ventricular, MCS – mechanical circulatory support, NPI – neurological pupil index, NSE – neuron specific enolase, PAC – pulmonal-arterial catheter, PAPI – pulmonary arterial pulsatility index, PCI – percutaneous coronary intervention, RV – right ventricular, S-100 – protein S-100, TTE – *trans*-thoracic echocardiography.

**Fig. 1:**
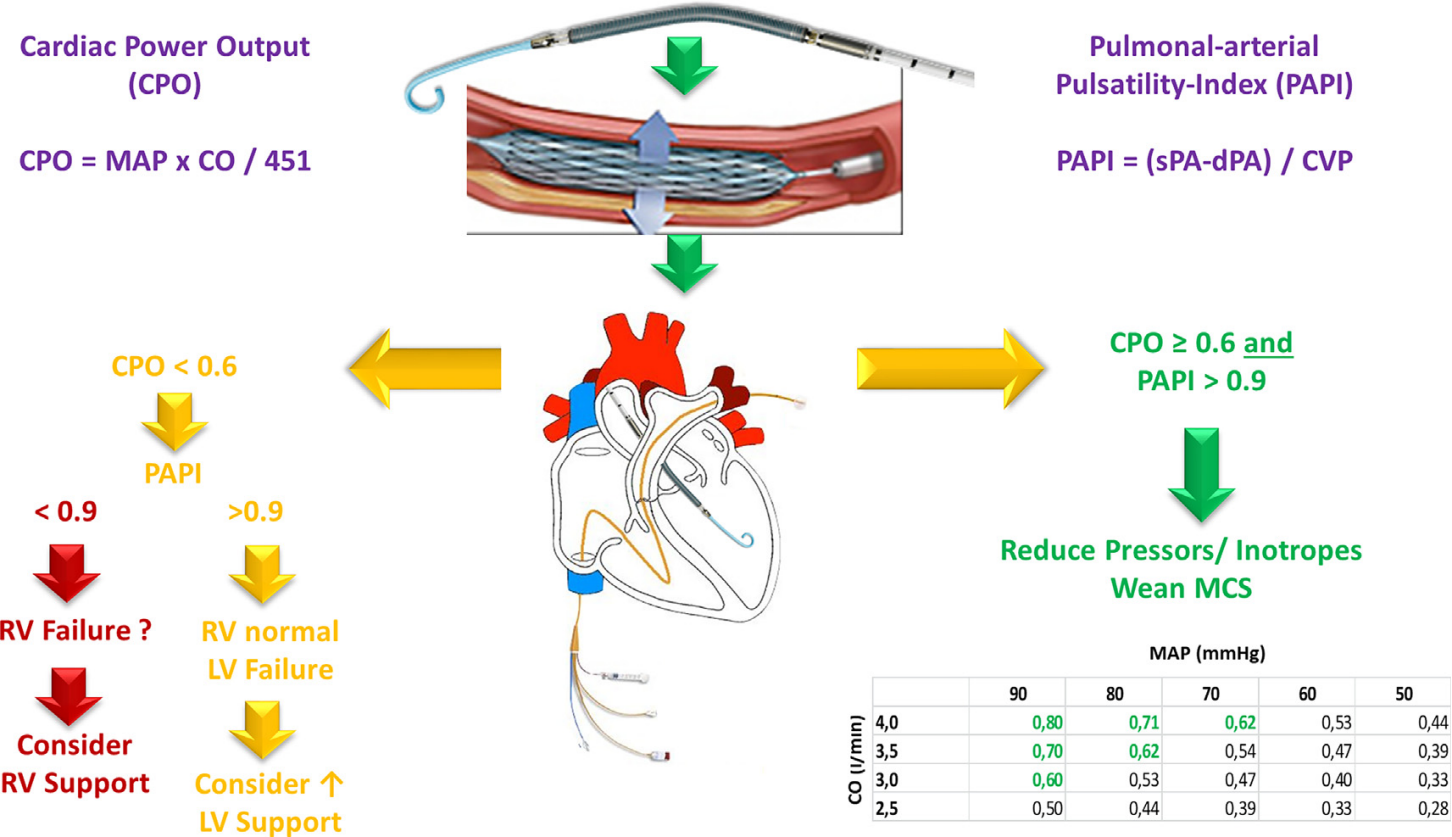
Patients are first stabilized in the catheterization laboratory with a micro-axial flow-pump and revascularized thereafter (as complete as possible) Upon arrival on the Cardiology Intensive care Unit (CICU) a pulmonary-arterial catheter (PAC) is placed for invasive monitoring during left-ventricular unloading. CO – cardiac output, CPO – cardiac power output, CVP – central venous pressure, LV – left ventricular, MAP – mean arterial pressure, MCS – mechanical circulatory support, PAPI – pulmonal-arterial pulsatility-index, s/dPA – systolic/diastolic pulmonary pressure, RV – right ventricular.

Care is subsequently delivered in a cardiology intensive care unit (CICU) equaling the highest level standards defined by the European Society of Cardiology’s Acute Cardiovascular Care Association^22^ and the American Heart Association^23^ that are staffed by dual-certified consultants in interventional cardiology and intensive care, to lower mortality of those patients.^24^ The sequence of haemodynamic guidance following initiation of LV unloading using an AFP with subsequent coronary revascularization in case of an AMI-CS is outlined in Fig. 1.

### Objectives

We report hospital outcomes over the first decade of HaCRA, providing real-world evidence from systematically managed OHCA and CS cohorts and benchmarking against contemporary trial populations.

## Methods

### HaCRA design

HaCRA was introduced in 2011 and first described in 2018.^12^ Based on experiences gained when applying the algorithm, we adapted the approach continuously. Because OHCA and AMI-CS share substantial overlap, a single structured algorithm was established for both entities. HaCRA standardizes early in-hospital management from emergency admission through computed tomography, cardiac catheterization, and transfer to CICU.

Initial assessment is performed by cardiologists and anaesthesiologists, led by a consultant dually certified in interventional cardiology and intensive care.^2^ The algorithm prioritizes exclusion of non-coronary causes of arrest, early coronary angiography and percutaneous intervention in all resuscitated and/or CS patients, and timely initiation of mechanical circulatory support (MCS) in patients with Society of Cardiovascular Angiography & Intervention (SCAI) stage C or worse CS.

Patients stabilized on device support undergo coronary angiography, with complete revascularization attempted whenever feasible.^14^, ^29^, ^30^ All OHCA patients who remain unconscious at admission receive active temperature management to induce therapeutic hypothermia for ≥24 h as post-resuscitation neuroprotection.^12^ A detailed protocol is available in the Supplement.

### Setting

HaCRA was implemented at Hannover Medical School as a standardized diagnostic and early treatment pathway for OHCA and CS. The goals were rapid recognition of life-threatening conditions, consistent application of hypothermia in OHCA, and early MCS in CS.

To enable systematic evaluation, two prospective registries were established. The *HAnnover COoling REgistry* (HACORE) includes all OHCA patients treated with hypothermia, and the *HAnnover Cardiac Unloading REgistry* (HACURE) includes all CS patients supported with an axial flow pump (AFP). From 2011 to September 2024, 946 consecutive OHCA patients were enrolled in HACORE and 506 CS patients in HACURE. Both registries allow prospective data capture and retrospective analysis, were approved by the Hannover Medical School ethics committee (HACORE #3567-2017, HACURE #3566-2017), and comply with the Declaration of Helsinki. HACORE follows Utstein reporting recommendations.^25^ All patients were admitted to the Hannover cardiac arrest center/CICU, a tertiary referral unit providing the highest level of care.^22^, ^23^

### Participants

For this analysis, all OHCA patients treated with hypothermia and all CS patients supported with an AFP were included. To ensure comparability with clinical trial populations, the HACORE cohort was screened according to *TTM-trial* inclusion and exclusion criteria.^4^ Adult OHCA patients with sustained ROSC at admission were eligible. Exclusion criteria were: ROSC > 240 min before admission, unwitnessed arrest with asystole as initial rhythm, acute intracranial hemorrhage or stroke, non-cardiac etiology, severe hypothermia (<30 °C), or profound cardiogenic shock.

### Variables

The primary outcome was 30 days in-hospital mortality. Patients were followed for the duration of their hospital stay. Functional neurological outcome was assessed using the cerebral performance category (CPC) scale.^26^, ^27^ CPC scores of 1 (good cerebral performance) or 2 (moderate cerebral disability) were classified as favorable outcome, scores of 3 (conscious but dependent/severe cerebral disability), 4 (coma), or 5 (death) as poor outcome.^26^, ^28^

### Data sources and measurements

Patient data, including the recommended Utstein style parameters,^25^ were extracted from the electronic hospital patient data management system into both registries. For patients transferred to one of three cooperating intensive care–rehabilitation facilities, discharge letters were collected by a trained study nurse to ensure consistent documentation of outcome data.

### Statistical methods

Continuous variables are reported as mean ± standard deviation (SD) for normally distributed data or as median with interquartile range (IQR) for skewed data. Categorical variables are presented as absolute numbers and percentages. Cumulative mortality was estimated using the Kaplan–Meier method. Analyses were performed with SPSS Statistics 24 (IBM, Armonk, NY, USA). Figures were created with GraphPad Prism 6.0 (GraphPad Software, La Jolla, CA, USA).

For the primary outcomes (in-hospital mortality and CPC), data completeness was 100 %, avoiding imputation and loss to follow-up.

## Results

### Patient characteristics

A total of 946 consecutive OHCA patients were included in HACORE. The mean age was 62 years, and 76 % were male. The initial rhythm at first medical contact was shockable in 59 %. OHCA was witnessed in 71 %, and bystander resuscitation was performed in 71 %. The mean time to ROSC was 27 min. Admission lactate was 7.8 mmol/L, and the mean SAPS II score at CICU admission was 52. MCS (ECMO and/or AFP) was required in 21 % of OHCA patients, and 24 % required renal replacement therapy. Not all patients receiving eCPR achieved sustained hemodynamics sufficient for CICU admission.

The HACURE registry included 506 consecutive CS patients treated with an AFP. Mean age was 60 years, with an equal sex distribution. The leading cause of CS was AMI (71 %), followed by cardiomyopathy (25 %). Admission lactate was 6.8 mmol/L, and the mean SAPS II score at CICU admission was 52. Prior cardiac arrest with ROSC had occurred in 37 % of patients, with a mean time to ROSC of 33 min. Renal replacement therapy was required in 50 % during the CICU stay. Detailed baseline characteristics by admission category (eCPR, OHCA with CS, isolated CS, and OHCA without CS) are presented in Table 1.

**Table 1:** Baseline data for patients being admitted with extra-corporeal cardiopulmonary resuscitation (eCPR) for ongoing arrest, haemodynamically stable out-of-hospital cardiac arrest (OHCA), cardiogenic shock treated with mechanical circulatory support (MCS), and those having OHCA and cardiogenic shock (CS) with MCS that were treated according to the Hannover Cardiac Resuscitation Algorithm (HaCRA).

**Demographics**	**eCPR**	**OHCA + AFP**	**OHCA w/o CS**	**CS AFP**
Number	66	140	733	188
Age – years (mean ± SD)	53 ± 12	59 ± 13	64 ± 15	61 ± 14
Male sex	49 (74 %)	119 (85 %)	539 (74 %)	96 (51 %)
**Cardiovascular disease burden**				
History of MI	4 (6 %)	16 (11 %)	123 (17 %)	48 (26 %)
History of PCI	4 (6 %)	19 (13 %)	96 (13 %)	71 (38 %)
History of CABG	2 (3 %)	4 (3 %)	68 (9 %)	17 (9 %)
History of stroke/TIA	2 (3 %)	13 (9 %)	73 (10 %)	13 (7 %)
History of peripheral artery disease	3 (5 %)	10 (7 %)	56 (8 %)	17 (9 %)
**Cardiovascular Risk factors**				
Smoking	26 (40 %)	51 (36 %)	241 (33 %)	76 (40 %)
Hypertension	29 (44 %)	71 (51 %)	405 (55 %)	108 (58 %)
Hyperlipidaemia	10 (15 %)	38 (27 %)	196 (27 %)	44 (23 %)
Diabetes	11 (17 %)	28 (20 %)	176 (24 %)	53 (28 %)
**Resuscitation parameters**				
ROSC, min (mean ± SD)	−	38 ± 27	24 ± 16	−
time-to-ECMO, min (mean ± SD)	85 ± 36	−	−	−
Shockable primary rhythm	38 (58 %)	103 (74 %)	399 (58 %)	−
Witnessed arrest	49 (74 %)	101 (72 %)	499 (68 %)	−
Bystander CPR	57 (86 %)	104 (74 %)	510 (70 %)	−
Cause of CS				
STEMI	−	80 (57 %)	−	73 (39 %)
NSTEMI	−	29 (21 %)	−	39 (22 %)
Cardiomyopathies, Myocarditis	−	31 (22 %)	−	76 (39 %)
**SCAI shock class**				
C	0 (0 %)	62 (44 %)	−	100 (53 %)
D	0 (0 %)	15 (11 %)	−	57 (30 %)
E	66 (100 %)	63 (45 %)	−	31 (16 %)
Coronary angiography	62 (94 %)	140 (100 %)	606 (83 %)	188 (100 %)
PCI	47 (71 %)	118 (84 %)	434 (59 %)	112 (60 %)
Micro-axial flow pump	36 (55 %)	140 (100 %)	−	188 (100 %)
vaECMO	66 (100 %)	59 (42 %)	−	84 (45 %)
Computed tomography	63 (95 %)	138 (99 %)	733 (100 %)	90 (48 %)
**In-hospital events**				
Renal replacement therapy	29 (44 %)	67 (48 %)	137 (19 %)	89 (48 %)
GUSTO moderate bleeding	60 (91 %)	112 (80 %)	85 (12 %)	120 (64 %)
GUSTO severe bleeding	3 (4 %)	17 (12 %)	7 (1 %)	22 (12 %)
**Admission parameters (mean ± SD)**				
SAPS II score	55 ± 9	53 ± 10	51 ± 12	51 ± 12
Systolic blood pressure (mmHg)	0 ± 0	101 ± 27	115 ± 48	100 ± 31
Left-ventricular end-diastolic pressure (mmHg)	−	27 ± 11	−	24 ± 6
Arterial lactate (mmol/l)	13.7 ± 2.4	9.1 ± 4.8	7.4 ± 4.2	4.6 ± 3.6
NT-proBNP (pg/ml)	823 ± 1925	7217 ± 10293	1845 ± 4673	14342 ± 11823
Glomerular filtration rate (ml/min)	74 ± 24	58 ± 26	64 ± 41	51 ± 24

AFP – axial flow-pump; CABG – coronary artery bypass graft; ECMO – extra-corporeal membrane oxygenation; GUSTO – Global Use of Streptokinase and t-PA for Occluded Coronary Arteries *(trial used for bleeding definition)*; ROSC – return-of-spontaneous circulation; NSTEMI – Non-ST-elevation myocardial infarction; PCI – percutaneous coronary intervention; SCAI – society for cardiovascular angiography & intervention *(for shock classification)*; STEMI – ST-elevation myocardial infarction; TIA – transient ischaemic attack.

### Overall mortality in resuscitated or cardiogenic shock patients at the CICU

**Fig. 2:**
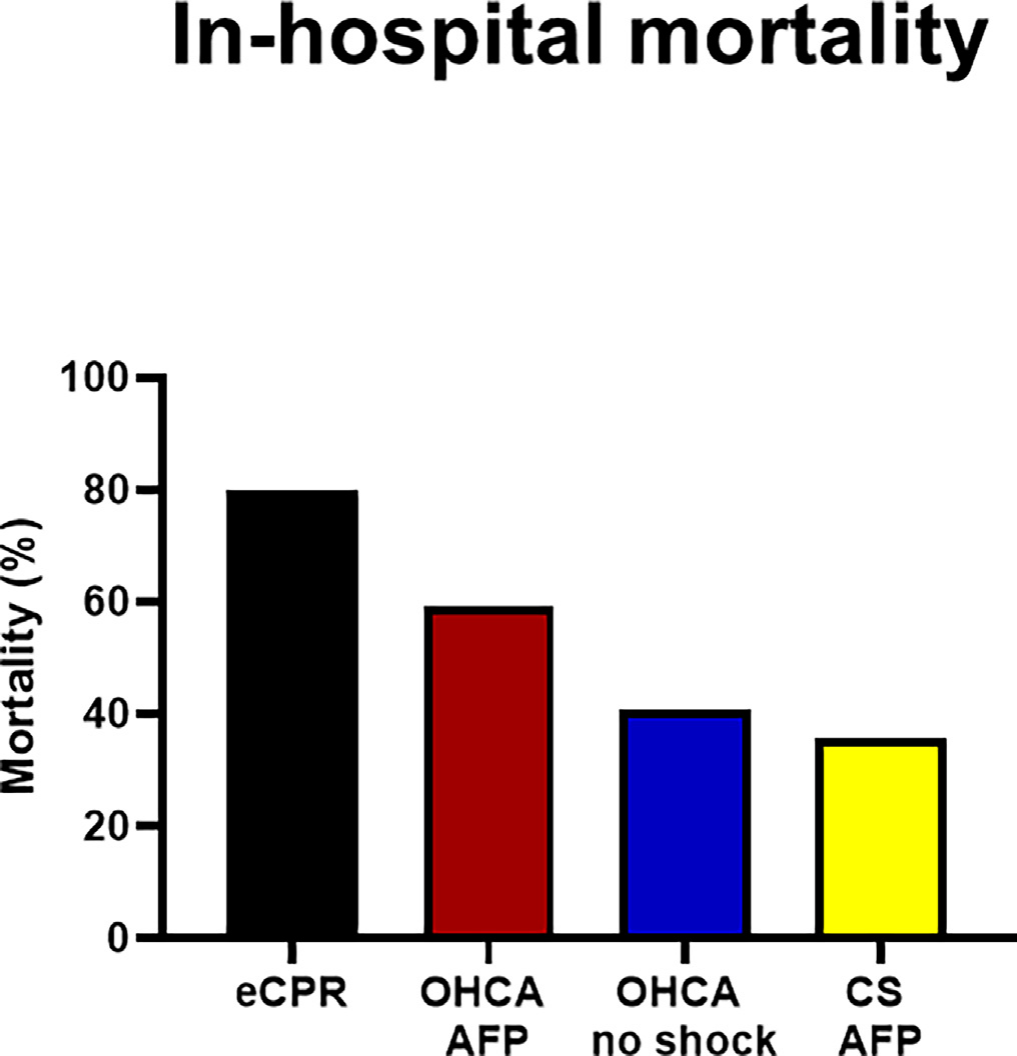
In-hospital mortality following admission for cardiac arrest or cardiogenic shock at our institution. AFP – axial flow-pump, CS – cardiogenic shock, eCPR – extra-corporeal cardiopulmonary resuscitation, MCS – mechanical circulatory support, OHCA – out-of-hospital cardiac arrest.

Fig. 2 shows in-hospital mortality rates for patients admitted with eCPR during ongoing arrest, hemodynamically stable OHCA, CS treated with MCS, and combined OHCA with CS requiring MCS under the HaCRA protocol.

**Fig. 3:**
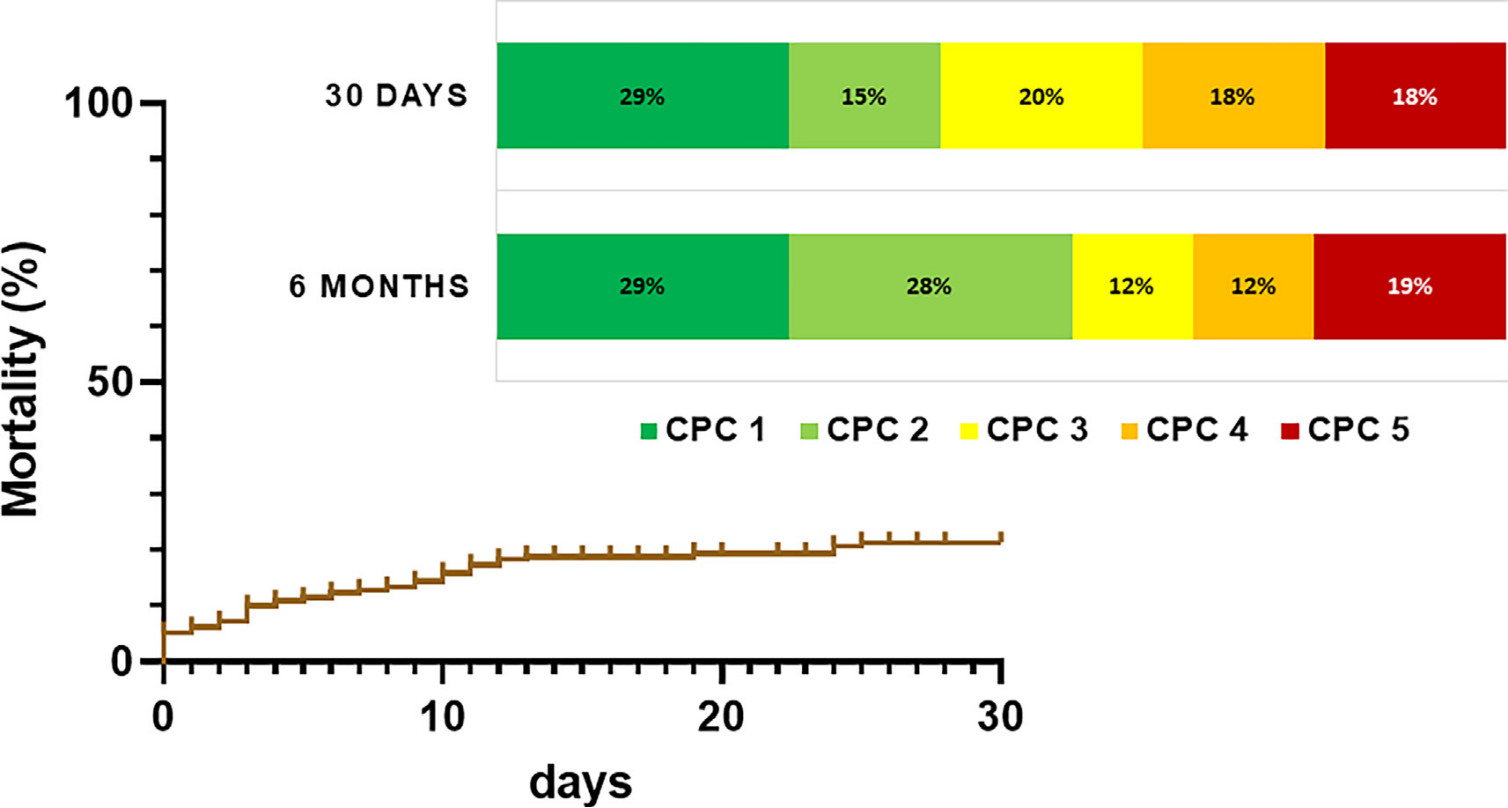
In-hospital mortality and functional neurological outcome in cardiac arrest-patients (observed, shockable, non-shock) was assessed by the cerebral performance category (CPC) at primary hospital discharge and 6-months follow-up.

Overall mortality in OHCA patients was 44 %. Mortality was 33 % in those with an initial shockable rhythm and 61 % in those with non-shockable rhythms. For comparability with contemporary trials, we selected patients fulfilling the TTM trial inclusion criteria. This subgroup had an in-hospital mortality of 44 %, predominantly in witnessed arrests with shockable rhythms and without CS. Restricting the analysis further to witnessed OHCA with shockable rhythm reduced mortality to 29 %. Among patients without MCS, mortality was 23 %, with mostly favorable neurological outcomes (Fig. 3).

**Fig. 4:**
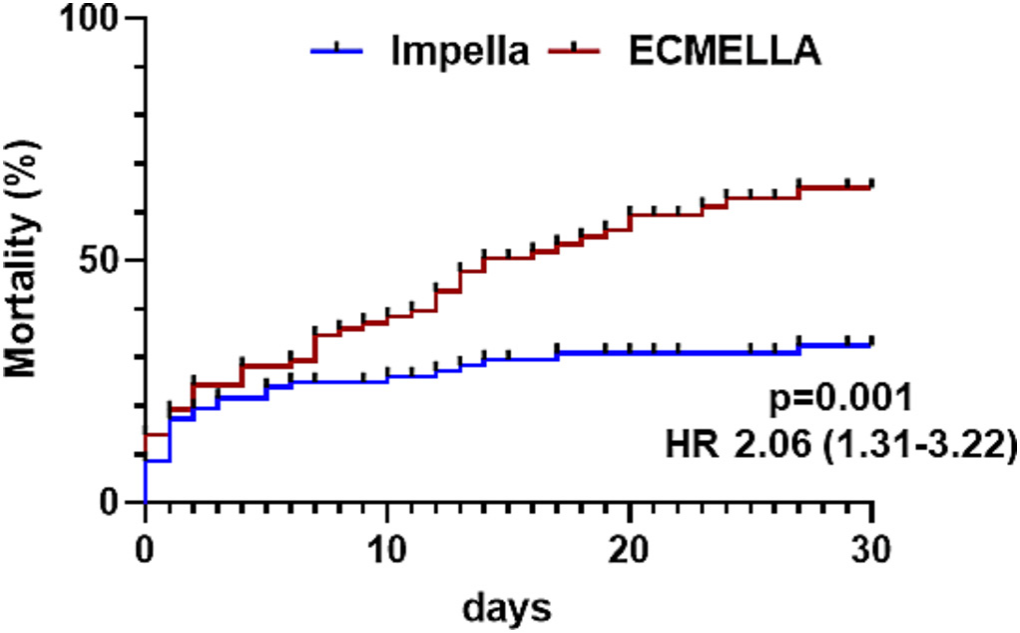
In-hospital mortality in cardiogenic shock is shown separately for patients being supported with an axial flow-pump alone (AFP) or in combination with extra-corporeal membrane oxygenation (ECMELLA).

In CS patients requiring MCS, overall in-hospital mortality was 52 % (48 % in AMI-related CS). Mortality varied substantially by type of support: patients treated with AFP alone had 35 % mortality, whereas those requiring combined LV unloading and VA-ECMO (ECMELLA) had 59 % mortality (Fig. 4). The higher mortality in the ECMELLA group likely reflects the more severe course of biventricular failure, which was the standard indication for escalation to this strategy.

## Discussion

Implementation of a standardized, multidisciplinary approach to OHCA and CS using HaCRA—including rapid emergency room screening, structured diagnostics (focused transthoracic echocardiography, computed tomography, and coronary angiography), protocolized therapeutic hypothermia (target 32 °C), and early hemodynamic stabilization via MCS—contributes to optimized patient outcomes.

HaCRA ensures that patients with CS (SCAI shock-class C or worse) and post-OHCA are managed by cardiologists trained in both interventional cardiology and intensive care.^2^ Physicians in training for on-call service complete a formal two-year CICU qualification under supervision of two dual-certified consultants. This integration of interventional cardiology and intensive care expertise provides comprehensive care that surpasses either discipline alone.

Placing CS and OHCA patients primarily in a dedicated CICU also ensures a high level of specialized nursing care. CICU nurses are trained in managing MCS, participate in the cardiac arrest team, and undergo repeated advanced cardiac life support (ACLS) training. Early activation of formal cardiac arrest or shock teams—including experts in cardiology, anesthesiology, cardiac surgery, perfusion, vascular interventions, and nursing—further strengthens patient management.^29^ HaCRA standardizes patient care in three phases: prehospital optimization, rapid diagnosis and treatment on admission, and structured intensive care.

The dedicated approach (also see Table S1) benefits not only patients at extreme risk upon admission but also those who deteriorate later (e.g., SCAI B to C/D) or initially stable patients after extensive anterior myocardial infarction. Close monitoring—including pulmonary artery catheterization, arterial lactate, echocardiography, and biomarkers—is essential for early detection of deterioration in patients with less severe shock.^30^

The overlap between OHCA and CS justified the use of a single algorithm to simplify and standardize early multidisciplinary care.^12^, ^31^ Selection criteria for eCPR candidates have evolved based on initial experience.^17^ Currently, eCPR is reserved for younger patients with witnessed arrest, shockable rhythm, and bystander CPR.

HaCRA deviates from some guideline recommendations when evidence is limited or neutral. For example, although guidelines recommended only chest X-ray after OHCA,^14^ we routinely performed early CT before ICU admission, identifying life-threatening conditions or complications in nearly 25 % of patients, enabling prompt targeted therapy.^32^ Early imaging reduces unnecessary transports and optimizes resource allocation.

Emerging data on MCS in CS supports the HaCRA pathway. In CS, stabilization often requires resolving the underlying cause or mechanical support.^33^ Early AFP placement is associated with lower mortality,^31^ particularly when initiated before revascularization.^21^, ^34^, ^35^ This knowledge was based on registry data in the absence of randomized trials during most of the HaCRA implementation.^31^, ^34^, ^36^ Nowadays, data from clinical trials support the initial use of an AFP in selected cases of AMI-CS,^5^, ^37^ as the DanGer-Shock trial was very selective in order to collect high-risk shock in large anterior AMI but to evade excess anoxic brain damage biasing the mortality outcome in resuscitated patients.^38^

The largest divergence from randomized trials was observed in witnessed OHCA with shockable rhythm, who comprise most TTM-trial patients. Mortality in this HaCRA cohort was lower than in the TTM-trial,^12^, ^39^ reflecting the benefit of therapeutic hypothermia and structured intensive care. Unlike the TTM-trials,^3^, ^4^ HaCRA does not mandate early prognostication or withdrawal of life support, consistent with current neurological recommendations.^40^ We also use much higher thresholds for neuromarkers such as neuron-specific enolase consistent with recommendations from neurological societies.^41^, ^42^ Post-CICU, comatose OHCA patients are transferred to specialized neuro-intensive care and rehabilitation, improving neurological outcomes over six months.

### Limitations

HaCRA is implemented in a tertiary university hospital, which may influence outcomes due to advanced stabilization and locally optimized care. This is a single-center, retrospective analysis without a control group, limiting generalizability. Nevertheless, key interventions such as endotracheal intubation, therapeutic hypothermia, early revascularization, complete revascularization, and MCS in CS remain supported by high-level evidence, even if trial conditions differ from our standardized CICU environment.

## Conclusion

HaCRA, applied in a tertiary CICU with integrated interventional cardiology and intensive care expertise, supports favorable outcomes in CS and OHCA. This experience highlights the value of developing cardiology intensive care as a formal subspecialty.

## CRediT authorship contribution statement

**Vera Garcheva:** Writing – original draft, Visualization, Supervision, Software, Methodology, Investigation, Formal analysis, Data curation, Conceptualization. **Tobias J. Pfeffer:** Writing – review & editing, Visualization, Validation, Resources, Methodology, Formal analysis. **Johann Bauersachs:** Writing – original draft, Validation, Supervision, Resources, Project administration, Methodology, Funding acquisition, Conceptualization. **Andreas Schäfer:** Writing – original draft, Visualization, Supervision, Software, Resources, Project administration, Methodology, Investigation, Funding acquisition, Formal analysis, Data curation, Conceptualization.

## Declaration of competing interest

The authors declare the following financial interests/personal relationships which may be considered as potential competing interests: ‘AS received lecture fees and honoraria from Abiomed, Amgen, AOP, Boehringer Ingelheim, BMS, Daiichi-Sankyo, Eli Lilly, Novartis, Pfizer, ZOLL as well as research support by Abiomed and Daiichi-Sankyo. JB received lecture fees and honoraria from Novartis, Abbott, Bayer, Pfizer, Boehringer Ingelheim, AstraZeneca, Cardior, CVRx, BMS, Amgen, Edwards, Roche, Zoll not related to this article; and research support for the department from Zoll, CVRx, Abiomed, Norgine, Roche, not related to this article. TJP received lecture fees and honoraria from Astra Zeneca. All other authors have no conflicts to declare.’.
