# Supplementary material for: Cadiology intensive care in patients with out-of-hospital cardiac arrest or cardiogenic shock
Source: Resusc Plus. 2025 Sep 30;26:101116. doi: 10.1016/j.resplu.2025.101116 (PMC12547704; doi:10.1016/j.resplu.2025.101116)
Supplement: Supplementary Table 1 [file mmc1.docx]

| **Focus** | **Standard Procedures**   - ER: Patient is received by a multidisciplinary team from CICU, anaesthesiology and ER nurses | |
| --- | --- | --- |
| **Sedation**  ***(CS/ OHCA)*** | - Sedation target: Ramsay 5-6 during therapeutic hypothermia and critical early phase of CS - Initially midazolam infusion 100 mg/50 ml (Max. Dose: 10 ml/h.) - Sufentanyl infusion 250 µg/50 ml (Max. Dose: 4 ml/h.) - If no adequate sedation on continuous EEG, add dexmedetomidine - AVOID additive narcotics when documented deep sedation in continuous EEG (F0)!! - Additive sodium thiopental on top of sufentanyl+midazolam only for seizures (→ EEG) being resistant to anti-epileptics | |
|  | ***OHCA***   - AVOID Propofol after OHCA!! - When haemodynamically stable during hypothermia, switch sedation to isoflurane + dexmedetomidine, stop midazolam. - Stop sedation when successfully rewarmed to body temperature of 36.0°C. | ***CS***   - AVOID volatile anaesthetics during early phase of CS due to vasoplegia |
| **Blood sampling**  ***(CS/ OHCA)*** | - On admission: Electrolytes (Sodium, Potassium, Calcium), Creatinine, blood urea, ALT, AST, Bilirubin, Troponin, Creatin kinase (-MB), LDH, CRP, blood cell count, global coagulation test, blood gases, central venous saturation - Routine sampling: 4 hourly blood gases - Bi-daily central venous saturation /pulmonary arterial saturation (if respective catheters in place) - Daily Sampling: Electrolytes (Sodium, Potassium, Calcium), Creatinine, blood urea, Troponin, Creatin kinase (-MB), LDH, CRP, blood cell count, global coagulation test - GDF-15 on admission | |
|  | ***OHCA***   - On admission and d1, d2, d3: NSE, S-100b, IL-6 | ***CS***   - On admission and d1, d2, d3, d4, d5: IL-6, NT-proBNP |
| **Ventilation**  ***(CS/ OHCA)*** | - Ventilation mode: BIPAP - Tidal volume: 6 ml/kg body weight, accepting higher ventilation rate - Peak pressure < 30 cm H_2_O - Target SpO_2_ 95%. pCO_2_: 40-45 mmHg | |
| **Therapeutic Hypothermia/ Temperature management**  ***(OHCA only)*** | - Indication: Any intra- or out-of-hospital cardiac arrest of any primary rhythm remaining unconscious after ROSC. - Cooling-catheter is used as first central line in ER, cath lab, or at latest after arrival on CICU. If patients go to the cath lab, a femoral cooling catheter is placed in the beginning to serve as central line as well - Urinary catheter with temperature probe - Rapid activation of cooling upon CICU arrival: target temperature 32°C, max. cooling,   - Maintain 32°C for 24h, rewarm with 0.25°C/h till temperature at 37°C   - In case of longer hypoxia/ longer no-flow/ primary supraglottic airway device maintain 32°C for 48h, rewarm with 0,1°C/h till temperature at 37°C - Cooling catheter remains for a minimum of 72 h after rewarming and maintains body temperature at 37°C (Fever mode). - Avoid fever after ROSC. If fever, use of antipyretics: Paracetamol i.v. and active cooling, when required change of normal central line to a cooling catheter - If shivering: Atracurium i.v. where required repetitive until reaching target temperature, sufentanyl/midazolam as standard analgosedation during induction of hypothermia, where required dexmedetomidine infusion. AVOID additive barbiturates for shivering under documented deep sedation!! - If shivering under normothermia when required surface counter warming - Hypothermia reduces heart rate: tolerate bradycardia until 30/min if lactate does not increase | |
| **Continuous Neuromonitoring**  ***(CS/ OHCA)*** | - 2-channel continuous EEG’s - Documentation of level of sedation and Burst-Suppression-Ratio 1x/ 12 hrs - Monitoring cerebral perfusion using rSO2   ***OHCA***   - Routine-EEGs during first 24h of hypothermia and during first 24h after reaching 37°C | |
| **Neurology**  ***(OHCA)*** | - Neurological examination: daily documentation of Babinski and brain stem reflexes, corneal, reaction to pain, coughing during tracheal suction, Glasgow-Coma-Scale. - Pupillary reaction every 2 h with Pupillometer (NPI) - Twice a day ultra sound of optical nerve (document diameter) - For seizures, epileptic or myoclonal state Lorazepam (2-4 mg slowly i.v.) - Neurologic consultation, EEG and imaging as required - Standardised ward round with neurology consultant twice weekly - Saturation for seizures with valproic acid (target level 50-100 mg/l) or Levetiracetam (500 mg 2 x daily i.v.; for renal insufficiency chose 50 mg Brivaracetam) | |
| **Diagnostic, Haemodynamics and Monitoring**  ***(CS/ OHCA)*** | - Chest X-ray after CICU admission only following central line (or PAC) - Bronchoscopy if imaging/history suggestive for aspiration - Implement invasive arterial pressure line - AVOID routine brachial artery lines except extraordinary circumstances. - Insert central line - If Norepinephrine >1.6 mg/h start arginine-vasopressin - Urinary catheter: Urine volume/h., measure body temperature | |
|  | ***OHCA***   - During ER admission: computed tomography of head, chest and abdomen - Target-MAP > 75 mmHg. Monitor cerebral oxygenation with near-infrared spectroscopy (rSO2).   - - If rSO2-signal decreases, elevate MAP, during rewarming eventually reduce or pause rewarming - Standardised extensive haemodynamic monitoring using PICCO over arterial sheath from cath lab in haemodynamically stable patients | ***CS***   - During ER admission: chest X-ray - Insert PAC for cardiogenic shock, high catecholamines, or if borderline haemodynamics - initial target-MAP: 65-70 mmHg (SVR 900 dyn), adopt for target-CPO >0.6 and PAPI >0.9 |
| **Coronary Angiography and PCI**  ***(CS/ OHCA)*** | ***OHCA***   - Coronary angiography during admission in case of ST-segement elevation or primary shockable rhythm | ***CS***   - All CS with undefined coronaries undergo coronary angiography - In case of AMI, complete revascularisation on MCS |
| **Mechanical**  **Circulatory Support**  ***(CS)*** | - Indication: Severely impaired LV-EF with   - Arterial lactate >2.0 mmol/l   - CPO <0.6 despite pressors/inotropes   - Unstable AMI   - LVEDP >15 mmHg - First MCS for LV failure: Impella CP - Consider vaECMO to escalate to ECMELLA if   - CPO persistant < 0.6 (alternatively Impella 5.5)   - PAPI < 0.9 (alternatively BiPELLA) | |
| **Platelet inhibition, Thrombus prophylaxis**  ***(CS/ OHCA)*** | - In case of visible thrombus formation during coronary angiography use glycoprotein-inhibitor (tirofiban) with bolus and infusion i.v. for 24h, NO therapeutic anticoagulation required for glycoprotein-inhibition!! - After CICU arrival and successful coronary stenting administer Prasugrel 60 mg via gastric tube, maintenance dose 10 mg/d independent of age or comorbidities; NO switch of P2Y12 inhibitors during hypothermia, unstable haemodynamics or fasting - Unfractionated heparin i.v., continuously infusing 10.000 IU / 50 ml at 2 ml/h for thrombus prophylaxis - If therapeutic anticoagulation is required use unfractionated heparin (target aPTT 40-60 sec, anti-Xa 0.5 IU/ml) - following stenting continue unfractionated heparin (PTT/anti-Xa guided) + Prasugrel and STOP acetylsalicylic acid | |
| **Glucose control**  ***(CS/ OHCA)*** | - Insulin infusion 50 IU short-acting insulin/50 ml - target glucose 8-10 mmol/l | |
| **Nutrition**  ***(CS/ OHCA)*** | - Start enteral feeding within 24 hours, if pressors/inotropes stable or falling - Parenteral feeding only when enteral caloric demand not achievable within 5 days - Duodenal tube at the day after CICU admission | |
| **Ulcer prophylaxis**  ***(CS/ OHCA)*** | - 1 x daily 40 mg pantoprazole i.v. in the evening | |
| **Empiric antibiotic therapy**  ***(CS/ OHCA)*** | - AVOID antibiotic therapy without anamnestic, bronchoscopic or radiologic suspicion for aspiration - Prior to any antibiotic therapy retain: pulmonary secretion, 2 x aerobe and anaerobe blood cultures, urine culture - With anamnestic, bronchoscopic or radiologic suspicion for aspiration or sepsis: Piperacillin/tazobactam 4.5 g 3x daily   - Additive administration of Azithromycin 500 mg 1x daily for 3 days only if suspicion for atypical pneumonia - Escalation for 2nd septic burst usually ceftazidime / fluoroquinolone, AVOID standardised switch to carbapenem - For severe sepsis (sepsis with septic-caused 2-fold organ failure)/ septic shock with unclear focus and possible nosocomial (multi-resistant) bacteria: Piperacillin/tazobactam 4.5 g 3x daily + Levofloxacin 500 mg 2x daily + Vancomycin 1 g 2 x daily - Adopt dependent on antibiogram - Re-evaluate antibiotic regime after 48-72 hours - Treatment duration: 5-7 days - Adapt antibiotic dose dependent on renal function | |

*aPTT - activated partial thromboplastin time, ALT – alanine transaminase, AMI – acute myocardial infarction, AST – aspartate transferase, BIPAP – Bilevel positive airway pressure, BIPELLA – BiVentricular Impella, CICU – Cardiology intensive care unit, CI – cardiac index, CO – cardiac output, CPO – cardiac power output, CRP – C-reactive protein, CS – cardiogenic shock, ECG – electro-cardiogram, ECMELLA – combination of veno-arterial ECMO and LV Impella, ECMO – extracorporeal membrane oxygenation, EEG – electro-encephalogram, ER – emergency room, GDF-15 – Growth-derived factor 15, IL-6 – Interleukin 6, LDH – lactate dehydrogenase, LV – left ventricular, LVE-EF – LV ejection fraction, LVEDP – LV end-diastolic pressure, MAP – mean arterial pressure, MCS – mechanical circulatory support, NPI - neurological pupil index, NSE – neuron specific enolase, NT-proBNP – N-terminal brain natriuretic peptide, OHCA – out-of-hospital cardiac arrest, PAC – pulmonal-arterial catheter, PAPI – pulmonary arterial pulsatility index, PCI – percutaneous coronary intervention, PCWP – post-capillary wedge-pressure, PICCO – pulse index continuous cardiac output, ROSC – return of spontaneous circulation rSO_2_ – forehead regional oxygen saturation, S-100 – protein S-100, SpO_2_ – oxygen saturation, SVR – systemic vascular resistance.*
