# Supplementary material for: Cadiology intensive care in patients with out-of-hospital cardiac arrest or cardiogenic shock
Source: Resusc Plus. 2025 Sep 30;26:101116. doi: 10.1016/j.resplu.2025.101116 (PMC12547704; doi:10.1016/j.resplu.2025.101116)
Supplement: Supplementary Data 1 [file mmc2.docx]

**Cadiology Intensive Care in Patients with Out-of-hospital Cardiac Arrest or Cardiogenic Shock**

Vera Garcheva^a^, MD, Tobias J. Pfeffer^a^, MD, Johann Bauersachs^a^, MD, Andreas Schäfer^a^, MD

**Hannover Cardiac Resuscitation Algorithm (HaCRA)**

The HaCRA approach was originally described in 2018[1]. Based on experiences gained when applying the algorithm, we adapted the approach continuously. Due to the overlap between out-of-hospital cardiac arrest (OHCA) and acute myocardial infarction-cardiogenic shock (AMI-CS) we chose to use a single algorithm for early in-hospital diagnostic workup and treatment in order to simplify the approach by individually changing multi-disciplinary teams. The algorithm covers the full time-span from hospital admission in the emergency room through computed-tomography and cardiac catheterization until admission to the cardiology intensive care unit (CICU). Patients are first screened and stabilised in the emergency room, where rapid rule-out of reversible causes of arrest as well as implementation of veno-arterial extracorporeal membrane oxygenation (vaECMO) for extracorporeal cardiopulmonary resuscitation (eCPR) can be performed by well-trained staff including a CICU team. Therefore, when patients arrive with ongoing resuscitation, an automated chest compression device (LUCAS, Jolife AB / Physio-Control, Lund, Sweden) is initiated and the patient is evaluated for eCPR. The process is based on our evaluation of the initial experience with eCPR[2] in line with reported larger-volume cohorts[3-5] and expert consensus[6, 7].

In patients with return of spontaneous circulation (ROSC), the overall treatment strategy in HaCRA includes primary screening by a team of cardiologists and anaesthesiologists, whereby the cardiology team leader is a board-certified interventional cardiologist as well as a board-certified intensivist[8]. It includes a 12-channel electrocardiogram, focused transthoracic echocardiography and a sonographic assessment similar to trauma patients in the emergency room[1, 9] as well as immediate endotracheal airway management in case of a supraglottic airway device[10].

CS is identified by visually severely impaired left-ventricular ejection fraction in transthoracic echocardiography in combination with either arterial lactate ≥2.0mmol/l, cardiac power output ≤0.6W, cardiac index <2.2 and/or pulmonary-capillary wedge- or left-ventricular end-diastolic pressure >15mmHg reflecting predominantly Society for Cardiovascular Angiography & Intervention (SCAI) schock-category C or worse[11]. In case of CS a mechanical circulatory support (MCS) device is implanted, which preferably is an axial flow-pump (AFP)[12, 13], followed by single-access coronary angiography[14, 15]. If ventilation is required in CS, non-invasive ventilation is preferred –when feasible- prior to MCS implantation and followed by endotracheal intubation on MCS to prevent haemodynamic deterioration during the process[9]. In biventricular failure, AFP is combined with vaECMO[16, 17]. Once haemodynamically stabilised on a device, coronary angiography is performed and as complete revascularisation as possible is attempted by percutaneous coronary intervention in patients with AMI-CS[^14^](#_ENREF_14)^,^[^29^](#_ENREF_29)^,^[^30^](#_ENREF_30). In cases of CS and OHCA, early circulatory support helps to compensate for the excessive CS-related risk[18].

All patients with either CS or OHCA without obvious non-cardiac causes of arrest (e.g. intracranial bleeding, suspected massive pulmonary embolism) are transferred to the catheterisation laboratory for diagnostic coronary angiography when ST-segment elevations are present or the first detected rhythm in arrest was ventricular fibrillation (pathological electrocardiogram, central illustration)[19-22]. Culprit-lesion revascularisation of haemodynamically relevant coronary stenosis is percutaneously performed in OHCA patients[23-25]. Prior to intensive care admission, all OHCA patients get a cranial, chest, and abdomen computed tomography to rule out any potentially life-threatening yet overseen causes of arrest or complications by the resuscitation efforts[26]. On CICU, MCS is guided by invasive haemodynamic measurement using a pulmonary arterial catheter with the intention to lower LV filling pressures <15 mmHg. Measured cardiac power output (target ≥0.6 W) and pulmonary arterial pulsatility index (target >0.9) are used to escalate and de-escalate LV and RV support as needed (Figure 1)[27, 28].

All OHCA patients remaining unconscious upon hospital admission receive active temperature management to induce therapeutic hypothermia as a mandatory post-resuscitation procedure for neuroprotection for at least 24 hours[1]. Omitting hypothermia is limited to patients with limited life-support by patient will, active critical bleeding due to abdominal or intracranial injuries, and those urgently requiring extensive life-saving surgical procedures. Therapeutic hypothermia was incorporated in the protocol based on the positive findings of the initial trials[29, 30]. While guidelines became more liberal following the publication of the “Targeted-temperature management (TTM)”-trials allowing a broader temperature range reaching from therapeutic hypothermia at 32°C-34°C through controlled normothermia at 36°C to fever prevention at <37.8°C[31, 32], we continued to use therapeutic hypothermia at 32°C[33], because the TTM1 and TTM2 trials had only investigated quite selected patient populations with quite debatable withdrawal-of-life-support decisions leading to a presumed neutral outcome[34-36]. For resuscitated patients, a cooling catheter is the first central line placed whenever one is required during the admission process[36]. To perform therapeutic hypothermia after CICU admission, an intravascular cooling catheter (preferred Coolguard Quattro®, ZOLL Medical, San Jose, CA, USA) is placed in the right femoral vein. Selection of the device is based on shorter time to target temperature and more strictly maintained target hypothermia in endovascular than surface cooling[36, 37]. An active cooling device was chosen to select and maintain a constant target temperature of 32°C for at least 24 hours followed by controlled rewarming (0.10°C-0.25°C per hour) and normothermia for another 72 hours[38, 39]. On CICU arrival, therapeutic hypothermia is immediately started in all unconscious OHCA survivors.

Sedation is initiated with midazolam and sufentanyl during emergent diagnostics and CICU admission and switched to dexetomidine and isoflurane/sevoflurane when patients are haemodynamically stable during hypothermia. Barbiturates and long-term use of midazolam are avoided to prevent accumulation, e.g. during hypothermia. As propofol might induce similar damage to the mitochondrial respiratory chain as caused by hypoxia[40-43], its use is limited to otherwise uncontrollable seizures. While such mitochondrial dysfunction was observed in myocardial ischemia and reperfusion injury experiments under propofol anaesthesia, no such observations were made on volatile anaesthetics[44]. Continuous 2-lead electroencephalogram and near-infrared spectroscopy for cerebral oxygenation are initiated in all unconscious patients to evaluate the outcome potential[45, 46] and guide vasopressor support in case of declining cerebral oxygenation (central illustration).

If coronary intervention is performed in OHCA and/or AMI-CS, routine antithrombotic treatment includes unfractionated heparin, intravenous acetylsalicylic acid and crushed prasugrel given via a nasogastric tube[47]. In case of required therapeutic anticoagulation (e.g. during MCS therapy), acetylsalicylic acid is given as a single bolus and paused afterwards as long as therapeutic anticoagulation is maintained in analogy to the usual approach to otherwise anticoagulated stented patients[48]. The full protocol is shown in supplementary table 1.

**References:**

[1] Akin M, Sieweke J-T, Zauner F, Garcheva V, Tongers J, Napp LC, et al. Mortality in patients with out-of-hospital cardiac arrest undergoing a standardised protocol including therapeutic hypothermia and routine coronary angiography - experience from the HAnnover COoling REgistry (HACORE). JACC Cardiovasc Interv. 2018;11:1811-20.

[2] Napp LC, Sanchez Martinez C, Akin M, Garcheva V, Kuhn C, Bauersachs J, et al. Use of extracorporeal membrane oxygenation for eCPR in the emergency room in patients with refractory out-of-hospital cardiac arrest. PLoS One. 2020;15:e0239777.

[3] Wengenmayer T, Rombach S, Ramshorn F, Biever P, Bode C, Duerschmied D, et al. Influence of low-flow time on survival after extracorporeal cardiopulmonary resuscitation (eCPR). Crit Care. 2017;21:157.

[4] Bougouin W, Dumas F, Lamhaut L, Marijon E, Carli P, Combes A, et al. Extracorporeal cardiopulmonary resuscitation in out-of-hospital cardiac arrest: a registry study. Eur Heart J. 2020;41:1961-71.

[5] Suverein MM, Lorusso R, van de Poll MCG. Extracorporeal CPR for Out-of-Hospital Cardiac Arrest. Reply. N Engl J Med. 2023;388:1916-7.

[6] Michels G, Wengenmayer T, Hagl C, Dohmen C, Bottiger BW, Bauersachs J, et al. Recommendations for extracorporeal cardiopulmonary resuscitation (eCPR): consensus statement of DGIIN, DGK, DGTHG, DGfK, DGNI, DGAI, DIVI and GRC. Clin Res Cardiol. 2019;108:455-64.

[7] Grunau B, Scheuermeyer FX, Stub D, Boone RH, Finkler J, Pennington S, et al. Potential Candidates for a Structured Canadian ECPR Program for Out-of-Hospital Cardiac Arrest. CJEM. 2016;18:453-60.

[8] Binzenhofer L, Gade N, Roden D, Saleh I, Lanz H, Sierra LV, et al. A contemporary training concept in critical care cardiology. Front Cardiovasc Med. 2024;11:1351633.

[9] Sieweke JT, Berliner D, Tongers J, Napp LC, Flierl U, Zauner F, et al. Mortality in patients with cardiogenic shock treated with the Impella CP microaxial pump for isolated left ventricular failure. Eur Heart J Acute Cardiovasc Care. 2020;9:138-48.

[10] Garcheva V, Sanchez Martinez C, Adel J, Pfeffer TJ, Akin M, Bauersachs J, et al. Increased rate of anoxic brain damage with laryngeal tube compared to endotracheal intubation in patients with shockable out-of-hospital cardiac arrest - Experience from the HAnnover COoling REgistry (HACORE). Resuscitation. 2024:110416.

[11] Baran DA, Grines CL, Bailey S, Burkhoff D, Hall SA, Henry TD, et al. SCAI clinical expert consensus statement on the classification of cardiogenic shock: This document was endorsed by the American College of Cardiology (ACC), the American Heart Association (AHA), the Society of Critical Care Medicine (SCCM), and the Society of Thoracic Surgeons (STS) in April 2019. Catheter Cardiovasc Interv. 2019.

[12] Moller JE, Engstrom T, Jensen LO, Eiskjaer H, Mangner N, Polzin A, et al. Microaxial Flow Pump or Standard Care in Infarct-Related Cardiogenic Shock. N Engl J Med. 2024;390:1382-93.

[13] Lüsebrink E, Kellnar A, Krieg K, Binzenhöfer L, Scherer C, Zimmer S, et al. Percutaneous transvalvular microaxial flow pump support in Cardiology. Circulation. 2022;145:1254-84.

[14] Wollmuth J, Korngold E, Croce K, Pinto DS. The Single-access for Hi-risk PCI (SHiP) technique. Catheter Cardiovasc Interv. 2020;96:114-6.

[15] Schäfer A, Alasnag M, Giacoppo D, Collet C, Rudolph TK, Roguin A, et al. High-risk percutaneous coronary intervention in patients with reduced left ventricular ejection fraction deemed not suitable for surgical revascularisation. A clinical consensus statement from the European Association of Percutaneous Cardiovascular Interventions (EAPCI) in collaboration with the ESC Working Group on Cardiovascular Surgery. EuroIntervention. 2025;21:22-34.

[16] Tongers J, Sieweke JT, Kuhn C, Napp LC, Flierl U, Rontgen P, et al. Early Escalation of Mechanical Circulatory Support Stabilizes and Potentially Rescues Patients in Refractory Cardiogenic Shock. Circ Heart Fail. 2020;13:e005853.

[17] Lusebrink E, Orban M, Kupka D, Scherer C, Hagl C, Zimmer S, et al. Prevention and treatment of pulmonary congestion in patients undergoing venoarterial extracorporeal membrane oxygenation for cardiogenic shock. Eur Heart J. 2020;41:3753-61.

[18] Sieweke JT, Akin M, Beheshty JA, Flierl U, Bauersachs J, Schäfer A. Unloading in refractory cardiogenic shock after out-of-hospital cardiac arrest due to acute myocardial infarction. Front Cardiovasc Med. 2021;8:704312.

[19] Spaulding CM, Joly LM, Rosenberg A, Monchi M, Weber SN, Dhainaut JF, et al. Immediate coronary angiography in survivors of out-of-hospital cardiac arrest. N Engl J Med. 1997;336:1629-33.

[20] Kern KB, Lotun K, Patel N, Mooney MR, Hollenbeck RD, McPherson JA, et al. Outcomes of Comatose Cardiac Arrest Survivors With and Without ST-Segment Elevation Myocardial Infarction: Importance of Coronary Angiography. JACC Cardiovasc Interv. 2015;8:1031-40.

[21] Garcia S, Drexel T, Bekwelem W, Raveendran G, Caldwell E, Hodgson L, et al. Early Access to the Cardiac Catheterization Laboratory for Patients Resuscitated From Cardiac Arrest Due to a Shockable Rhythm: The Minnesota Resuscitation Consortium Twin Cities Unified Protocol. J Am Heart Assoc. 2016;5: e002670.

[22] Garcheva V, Akin M, Adel J, Sanchez Martinez C, Bauersachs J, Schafer A. High rate of critical coronary stenosis in comatose patients with Non-ST-elevation out-of-hospital cardiac arrest (NSTE-OHCA) undergoing therapeutic hypothermia-Experience from the HAnnover COoling REgistry (HACORE). PLoS One. 2021;16:e0251178.

[23] Dumas F, Bougouin W, Geri G, Lamhaut L, Rosencher J, Pene F, et al. Emergency Percutaneous Coronary Intervention in Post-Cardiac Arrest Patients Without ST-Segment Elevation Pattern: Insights From the PROCAT II Registry. JACC Cardiovasc Interv. 2016;9:1011-8.

[24] Millin MG, Comer AC, Nable JV, Johnston PV, Lawner BJ, Woltman N, et al. Patients without ST elevation after return of spontaneous circulation may benefit from emergent percutaneous intervention: A systematic review and meta-analysis. Resuscitation. 2016;108:54-60.

[25] Noc M, Fajadet J, Lassen JF, Kala P, MacCarthy P, Olivecrona GK, et al. Invasive coronary treatment strategies for out-of-hospital cardiac arrest: a consensus statement from the European association for percutaneous cardiovascular interventions (EAPCI)/stent for life (SFL) groups. EuroIntervention. 2014;10:31-7.

[26] Adel J, Akin M, Garcheva V, Vogel-Claussen J, Bauersachs J, Napp LC, et al. Computed-Tomography as First-line Diagnostic Procedure in Patients With Out-of-Hospital Cardiac Arrest. Front Cardiovasc Med. 2022;9:799446.

[27] Gorgis S, Gupta K, Lemor A, Bentley D, Moyer C, Mc RT, et al. Impact of Right Ventricular Dysfunction on Outcomes in Acute Myocardial Infarction and Cardiogenic Shock: Insights from the National Cardiogenic Shock Initiative. J Card Fail. 2024;30:1275-84.

[28] Basir MB, Kapur NK, Patel K, Salam MA, Schreiber T, Kaki A, et al. Improved outcomes associated with the use of shock protocols: updates from the National Cardiogenic Shock Initiative. Catheter Cardiovasc Interv. 2019;93:1173-83.

[29] Hypothermia after Cardiac Arrest Study G. Mild therapeutic hypothermia to improve the neurologic outcome after cardiac arrest. N Engl J Med. 2002;346:549-56.

[30] Bernard SA, Gray TW, Buist MD, Jones BM, Silvester W, Gutteridge G, et al. Treatment of comatose survivors of out-of-hospital cardiac arrest with induced hypothermia. N Engl J Med. 2002;346:557-63.

[31] Nolan JP, Sandroni C, Bottiger BW, Cariou A, Cronberg T, Friberg H, et al. European Resuscitation Council and European Society of Intensive Care Medicine guidelines 2021: post-resuscitation care. Intensive Care Med. 2021;47:369-421.

[32] Panchal AR, Bartos JA, Cabanas JG, Donnino MW, Drennan IR, Hirsch KG, et al. Part 3: Adult Basic and Advanced Life Support: 2020 American Heart Association Guidelines for Cardiopulmonary Resuscitation and Emergency Cardiovascular Care. Circulation. 2020;142:S366-S468.

[33] Lopez-de-Sa E, Juarez M, Armada E, Sanchez-Salado JC, Sanchez PL, Loma-Osorio P, et al. A multicentre randomized pilot trial on the effectiveness of different levels of cooling in comatose survivors of out-of-hospital cardiac arrest: the FROST-I trial. Intensive Care Med. 2018;44:1807-15.

[34] Dankiewicz J, Cronberg T, Lilja G, Jakobsen JC, Levin H, Ullen S, et al. Hypothermia versus Normothermia after Out-of-Hospital Cardiac Arrest. N Engl J Med. 2021;384:2283-94.

[35] Nielsen N, Wetterslev J, Cronberg T, Erlinge D, Gasche Y, Hassager C, et al. Targeted temperature management at 33 degrees C versus 36 degrees C after cardiac arrest. N Engl J Med. 2013;369:2197-206.

[36] Schafer A, Bauersachs J, Akin M. Therapeutic Hypothermia Following Cardiac Arrest After the TTM2 trial - More Questions Raised Than Answered. Curr Probl Cardiol. 2021:101046.

[37] Deye N, Cariou A, Girardie P, Pichon N, Megarbane B, Midez P, et al. Endovascular Versus External Targeted Temperature Management for Patients With Out-of-Hospital Cardiac Arrest: A Randomized, Controlled Study. Circulation. 2015;132:182-93.

[38] Ibanez B, James S, Agewall S, Antunes MJ, Bucciarelli-Ducci C, Bueno H, et al. ESC Scientific Document Group. 2017 ESC guidelines for the management of acute myocardial infarction in patients presenting with ST-segment elevation. Eur Heart J. 2018;39:119-77.

[39] Donnino MW, Andersen LW, Berg KM, Reynolds JC, Nolan JP, Morley PT, et al. Temperature Management After Cardiac Arrest: An Advisory Statement by the Advanced Life Support Task Force of the International Liaison Committee on Resuscitation and the American Heart Association Emergency Cardiovascular Care Committee and the Council on Cardiopulmonary, Critical Care, Perioperative and Resuscitation. Circulation. 2015;132:2448-56.

[40] Rigoulet M, Devin A, Averet N, Vandais B, Guerin B. Mechanisms of inhibition and uncoupling of respiration in isolated rat liver mitochondria by the general anesthetic 2,6-diisopropylphenol. Eur J Biochem. 1996;241:280-5.

[41] Branca D, Roberti MS, Lorenzin P, Vincenti E, Scutari G. Influence of the anesthetic 2,6-diisopropylphenol on the oxidative phosphorylation of isolated rat liver mitochondria. Biochem Pharmacol. 1991;42:87-90.

[42] Berndt N, Rosner J, Haq RU, Kann O, Kovacs R, Holzhutter HG, et al. Possible neurotoxicity of the anesthetic propofol: evidence for the inhibition of complex II of the respiratory chain in area CA3 of rat hippocampal slices. Arch Toxicol. 2018;92:3191-205.

[43] Finsterer J, Frank M. Propofol Is Mitochondrion-Toxic and May Unmask a Mitochondrial Disorder. J Child Neurol. 2016;31:1489-94.

[44] Lotz C, Stumpner J, Smul TM. Sevoflurane as opposed to propofol anesthesia preserves mitochondrial function and alleviates myocardial ischemia/reperfusion injury. Biomed Pharmacother. 2020;129:110417.

[45] Akin M, Sieweke JT, Garcheva V, Martinez CS, Adel J, Plank P, et al. Additive Impact of Interleukin 6 and Neuron Specific Enolase for Prognosis in Patients With Out-of-Hospital Cardiac Arrest - Experience From the HAnnover COoling REgistry. Front Cardiovasc Med. 2022;9:899583.

[46] Akin M, Garcheva V, Sieweke J-T, Adel J, Sanchez Martinez C, Bauersachs J, et al. Neuromarkers and neurological outcome in patients with out-of-hospital cardiac arrest treated with therapeutic hypothermia - Experience from the HAnnover COoling REgistry (HACORE). PLoS ONE. 2021;16:e0245210.

[47] Flierl U, Röntgen P, Zauner F, Tongers J, Bauersachs J, Schäfer A. Efficacy of platelet inhibition with prasugrel in patients with acute myocardial infarction undergoing therapeutic hypothermia after cardiopulmonary resuscitation. Thromb Haemost. 2016;115:960-8.

[48] Schäfer A, Flierl U, Bauersachs J. Anti-thrombotic strategies in patients with atrial fibrillation undergoing PCI. Clin Res Cardiol. 2021;110:759-74.
